# Connexin Mutations and Hereditary Diseases

**DOI:** 10.3390/ijms23084255

**Published:** 2022-04-12

**Authors:** Yue Qiu, Jianglin Zheng, Sen Chen, Yu Sun

**Affiliations:** 1 Institute of Otorhinolaryngology, Tongji Medical College, Huazhong University of Science and Technology, Wuhan 430022, China; 2020xh0034@hust.edu.cn; 2 Department of Neurosurgery, Huazhong University of Science and Technology, Wuhan 430022, China; 2020508093@hust.edu.cn

**Keywords:** connexin, gap junction, gene mutation, hereditary deafness, congenital cataract, congenital heart diseases, hereditary skin diseases

## Abstract

Inherited diseases caused by connexin mutations are found in multiple organs and include hereditary deafness, congenital cataract, congenital heart diseases, hereditary skin diseases, and X-linked Charcot–Marie–Tooth disease (CMT1X). A large number of knockout and knock-in animal models have been used to study the pathology and pathogenesis of diseases of different organs. Because the structures of different connexins are highly homologous and the functions of gap junctions formed by these connexins are similar, connexin-related hereditary diseases may share the same pathogenic mechanism. Here, we analyze the similarities and differences of the pathology and pathogenesis in animal models and find that connexin mutations in gap junction genes expressed in the ear, eye, heart, skin, and peripheral nerves can affect cellular proliferation and differentiation of corresponding organs. Additionally, some dominant mutations (e.g., Cx43 p.Gly60Ser, Cx32 p.Arg75Trp, Cx32 p.Asn175Asp, and Cx32 p.Arg142Trp) are identified as gain-of-function variants in vivo, which may play a vital role in the onset of dominant inherited diseases. Specifically, patients with these dominant mutations receive no benefits from gene therapy. Finally, the complete loss of gap junctional function or altered channel function including permeability (ions, adenosine triphosphate (ATP), Inositol 1,4,5-trisphosphate (IP3), Ca^2+^, glucose, miRNA) and electric activity are also identified in vivo or in vitro.

## 1. Introduction

Connexin-formed gap junctions are clusters of channels for intercellular communication that function by exchanging ions, small RNAs, nutrients, antioxidants, and second messengers between adjacent cells and play an important role in the coordination of cellular electrical response and metabolism in multicellular organisms [1]. Except for several fully differentiated cell types such as red blood cells, skeletal muscle cells and circulating lymphocytes, most cells in different tissues are coupled by gap junctions that function in intercellular communication [2]. Connexin problems are known to be involved in various diseases. Among them, inherited diseases caused by connexin mutations are found in multiple organs and include hereditary deafness, congenital cataract, congenital heart diseases, hereditary skin diseases, and X-linked Charcot–Marie–Tooth disease (CMT1X) [3]. Although the tissue types of the organs involved are completely different, these diseases may share the same pathogenesis, namely intercellular communication disorder.

To date, twenty-one connexins have been identified in humans and twenty connexins have been identified in mice; these connexins are designated as CX (human) or Cx (murine) followed by a number that indicates their molecular mass (kilodalton, kDa) (Table 1) [4,5]. The corresponding genes are identified by “*GJ*” (human) or “*Gj*” (murine) prefixes (Table 1). Connexins share a common structure that consists of four transmembrane segments (M1–M4), two extracellular loops (E1–E2), one cytoplasmic loop (CL), an intracellular amino-terminal (NT), and a carboxy-terminal (CT) [6]. Sequence alignment has identified that highly conserved residues are mainly located at the extracellular loops (E1–E2) and transmembrane segments (M1–M4), while the CL and CT vary greatly among different connexins [2,7,8]. Recent studies have further revealed the characteristic structure of different connexin-based channels using X-ray and cryo-electron microscopy (cryo-EM) techniques. There are no substantial structural differences (except for slight rotation and tilting) in the M1–M4 helices, while notable differences were observed in the NT helices and E1–E2 among the Cx31.3 hemi-channel, Cx46/Cx50 gap junction channel, and Cx26 hemi-channel or gap junction channel. These differences may be associated with channel docking, gating, and permeability [9,10,11,12].

Gap junctions are formed by two docked connexons located on adjacent plasma membranes. A connexon, also called a hemichannel, is a hexameric complex formed by connexins. In addition to docking to form gap junctions between adjacent cell membranes, connexons (hemichannels) also act as isolated, non-junctional transmembrane channels for intracellular–extracellular exchange [13]. There are four expression patterns of gap junctions: homomeric-homotypic, homomeric-heterotypic, heteromeric-homotypic, and heteromeric-heterotypic. A homomeric connexon consists of six identical connexins, while a heteromeric connexon contains different connexins. Two identical connexons form a homotypic gap junction and two different connexons form a heterotypic gap junction. Thousands of clustered gap junction channels form plaques [14].

**Figure 1:**
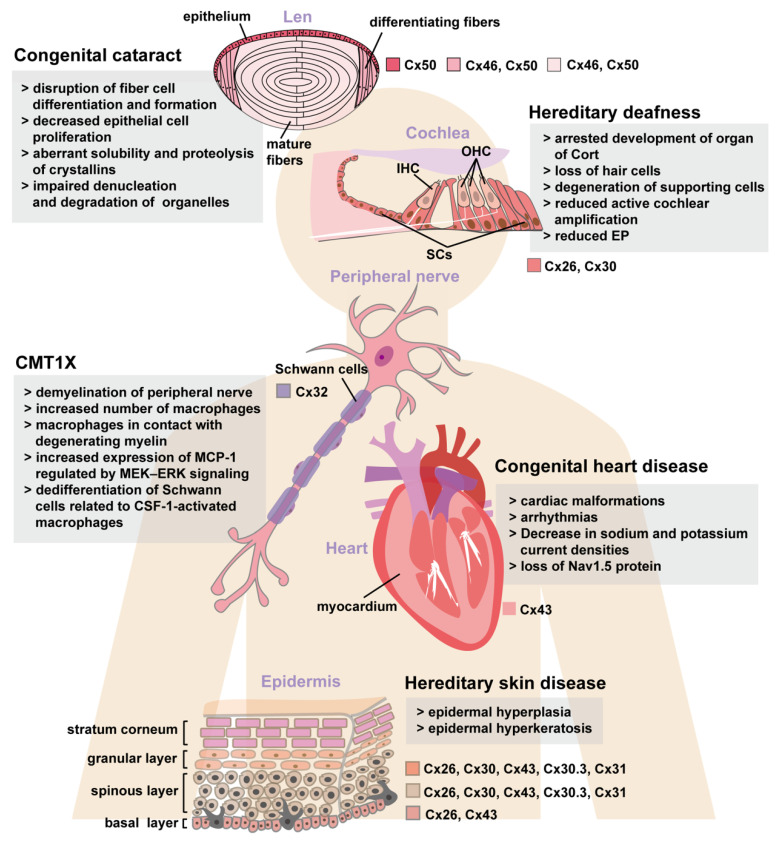
Main pathophysiological changes associated with human hereditary deafness, congenital cataract, congenital heart diseases, hereditary skin diseases, and CMT1X found in knock-in and knockout mouse models. EP: endolymphatic potential, OHC: outer hair cell, IHC: inner hair cell, SCs: supporting cells, MCP-1: monocyte chemoattractant protein-1, CSF-1: colony-stimulating factor 1.

Because the structures of different connexins are highly homologous and the functions of gap junctions formed by different connexins are similar [1,14], hereditary diseases induced by connexin mutations may share the same pathogenesis. The role of connexin mutations in these hereditary diseases cannot be easily revealed through cellular experiments for other no connexin-expressed cells also responding to connexin mutation mediated cellular stress. For example, genetic knockout of Cx26 in supporting cells (SCs) of the inner ear can induce Cx26-negative hair cell (HC) death. It is unclear why Cx26 deletion results in loss of HCs rather than SCs (nearly all SCs express Cx26) in *Gjb2*-related hereditary deafness. Additionally, it is unclear why some Cx26 mutations only result in non-syndromic hereditary deafness while others lead to deafness accompanied with skin diseases. Similarly, it is unknown why some Cx43 mutations result in oculodentodigital dysplasia (ODDD) while others cause heart disease only. The mechanisms underlying these observations need to be elucidated under pathophysiological conditions in vivo. Thus, in this review, we analyze and compare the similarities and differences of the pathology and pathogenesis of hereditary diseases including hereditary deafness, congenital cataract, congenital heart diseases, hereditary skin diseases, and CMT1X through related animal models (Figure 1 and Table 1 and Table 2). We also summarize common cellular mechanisms of connexin mutations in an effort to foster new ideas for the diagnosis and treatment of these diseases.

## 2. Hereditary Diseases and Connexins

### 2.1. Hereditary Deafness

#### 2.1.1. Connexin Mutations and Hereditary Deafness

Mutations in gene encoding connexins are the most common etiologic factors of hereditary deafness [67]. Multiple connexin genes have been found in the mammalian cochlea. *Gjb2* (encoding Cx26) and *Gjb6* (encoding Cx30) are the predominant connexin genes that are widely expressed in SCs of the cochlear epithelium and connective tissues of the inner ear [68,69,70,71]. *Gjb3*, encoding Cx31, has been found to be expressed in type III fibrocytes below the spiral prominence. *Gja1* (encoding Cx43) was expressed transiently in the connective tissues and immature sensory epithelium of mice [72]. *Gjb1* (encoding Cx32) was expressed in melanocytes of the murine inner ear [73].

The majority of more than 300 *GJB2* (encoding Cx26) gene mutations account for about 50% of all cases of autosomal recessive non-syndromic hereditary deafness. Additionally, several *GJB2* gene mutations cause autosomal dominant non-syndromic or syndromic deafness. *GJB2*-related deafness is congenital or delayed and is mild to profound [14,74,75,76,77]. These variants include missense, nonsense, and frameshift mutations (http://www.hgmd.cf.ac.uk/ac/index.php (accessed on 8 April 2022)). In Europe, the most prevalent *GJB2* mutation is c.35delG, while c.167delT is the most frequent variant in Ashkenazi Jewish families [78,79]. In China, the predominant form of *GJB2*-related deafness involves the c.235delC mutation [80,81]. The severity of *GJB2*-related deafness is widely diverse, even among siblings with the same genotype [82]. However, there are some correlations when variants are classified according to the severity of their molecular effects. Two truncating (T) mutations (mutations leading to premature stop codons, such as c.35delG, c.167delT, or c.235delC) resulted in more severe hearing loss, while biallelic non-truncating (NT) mutations (such as p.Met34Thr or p.Val37Ile) were more likely to cause mild to moderate deafness [83,84]. Although the mean auditory thresholds of subjects carrying p.Val37Ile are much lower than those carrying biallelic truncating variants, a small number of patients still show severe hearing loss [85].

*GJB6* (encoding Cx30) gene mutations also cause hereditary deafness that ranges from moderate to profound [86]. To date, over 20 mutations in the *GJB6* gene have been reported (http://www.hgmd.cf.ac.uk/ac/index.php (accessed on 8 April 2022)). These mutations mainly lead to autosomal recessive non-syndromic deafness. In 1999, Grifa et al. reported a missense mutation in the *GJB6* gene (Cx30 p.Thr5Met) that leads to autosomal dominant hearing loss. The degree of deafness ranges from moderate to profound and can be progressive [86].

Notably, large deletions in the *GJB2* or *GJB6* gene correspond with a monogenic or digenic mode of inheritance and induce deafness either in a homozygous or heterozygous state [87,88,89]. Furthermore, *GJC3* (encoding Cx30.2/31.3), *GJB3* (encoding Cx31), *GJB1* (encoding Cx32), and *GJA1* (encoding Cx43) gene mutations have been reported to cause non-syndromic deafness [90,91,92,93,94,95,96]. Because cochlear samples are difficult to obtain from patients, transgenic animal models are essential to pathogenesis research of hereditary deafness caused by connexin mutations.

#### 2.1.2. Mouse Models for Human Hereditary Deafness

In order to further explore the pathological mechanism of deafness caused by gap junction mutations, various gene knockout and knock-in models have been established. In general, the knock-out model can simulate the pathology of truncated mutations, while the knock-in model is used to study the pathophysiological changes of the corresponding missense mutations.

##### *Gjb2*-Related Deafness Models

To date, only three Cx26 mutation knock-in mouse models (CAG-Cre;Cx26^+/floxR75W^, pgk-Cre;Cx26^+/floxS17F^, and Cx26^V37I^) have been successfully established [43,44]. The Cx26 p.Arg75Trp mutation can cause autosomal dominant deafness and palmoplantar keratoderma, and in vitro research evidence has shown that this mutant protein interferes with gap junction function [97,98]. CAG-Cre;Cx26^+/floxR75W^ mice show congenital deafness, malformation of SCs, collapse of the tunnel of Corti (TC), and degeneration of HCs [43]. Further research has shown that microtubules are reduced in certain SCs of this mouse line, and programmed cell death in the greater epithelial ridge is delayed by p.Arg75Trp mutation [99,100]. In humans, the Cx26 p.Ser17Phe mutation has been found to cause keratitis-ichthyosis-deafness (KID) syndrome in an autosomal dominant manner [101]. Homozygous pgk-Cre;Cx26^+/floxS17F^ mice cannot survive, and heterozygotes show moderate deafness and a significant reduction in endolymphatic potential (EP) [44]. However, there is currently no published literature on the inner ear pathology of this mutation. Mice with a homozygous p.Val37Ile mutation develop progressive, mild-to-moderate hearing loss over 4–9 months with a minor loss of HCs. No significant morphological abnormalities were observed in the cochlea of this mouse line [102].

In addition, many Cx26 knockout mouse models have been reported for mechanism research in vivo. These models use promoters that are widely expressed in the inner ear, which act to knock out the *Gjb2* gene in as many regions of the inner ear as possible. The conditional knockout mice (Otog-Cre;Cx26^LoxP/LoxP^ and Sox10-Cre;Cx26^LoxP/LoxP^) in which Cx26 is specifically knocked out in the cochlear epithelium show significant hearing loss with the cell death of SCs and HCs [45,46]. However, three other Cx26 knockout strains (Pax2-Cre;Cx26^LoxP/LoxP^, Foxg1-Cre;Cx26^LoxP/LoxP^, and Rosa26-CreER;Cx26^LoxP/LoxP^) all exhibit arrested development of the organ of Corti before sensory epithelial cell loss [47], which indicates that Cx26 is essential for normal development and maturation of the organ of Corti.

Another research strategy is to modify the timing of *Gjb2* gene knockout. In timed conditional Cx26 null models, severe deafness and developmental arrest were observed in the early knockout group after birth [103,104,105]. Knockout of cochlear Cx26 after postnatal day 8 (P8) does not cause significant deafness or developmental abnormalities [105,106]. These studies indicate that Cx26 plays an important role in the maturation process of the organ of Corti prior to the onset of hearing;Cx26 reduction in more mature cochleae had a much weaker effect in damaging hearing [107].

The cell-specific knockout strategy is also a common way to study *Gjb2*-related deafness. In Lgr5-CreER;Cx26^LoxP/LoxP^ and Prox1-CreER;Cx26^LoxP/LoxP^ mice, Cx26 is specifically knocked out in SCs of the organ of Corti. In Lgr5-CreER;Cx26^LoxP/LoxP^ mice, Cx26 is knocked out in the third row of Deiter’s cells (DCs). This mouse model shows late-onset high-frequency deafness with corresponding outer hair cell (OHC) loss. However, the Cx26-null DCs show larger phalangeal processes with limited ultrastructure changes [49]. These findings indicate that Cx26-null DCs cannot maintain the survival of OHCs. Prox1-CreER;Cx26^LoxP/LoxP^ mice, in which Cx26 is knocked out in DCs and outer pillar cells (OPCs), exhibit severe hearing loss at high frequencies without apparent hair cell degeneration. OHC electromotility is influenced and active cochlear amplification is reduced, which is believed to lead to hearing loss [48,108]. Using the same mouse model, a subsequent study showed that conditional Cx26 knock-out in DCs and OPCs desensitizes mid-to-high frequency distortion product otoacoustic emissions (DPOAEs) and active basilar membrane (BM) responses [109]. Notably, none of the above knockout model studies addressed the developmental disorders of the inner ear.

##### *Gjb6*-Related Deafness Models

Cx30 may not be essential for normal hearing, and deafness may be due to a significant reduction in the protein level of Cx26. Cx30^T5M/ T5M^ and Cx30^−/−^ mice both show hearing loss with about 70–90% decrease in protein levels of Cx26. However, BAC^Cx26^;Cx30^−/−^ mice, in which protein expression of Cx26 returns to normal levels, exhibit normal hearing [54,55]. Moreover, pgk-Cre;Cx30^flox/flox^ mice, which have about 50% decrease in protein level of Cx26, also have normal hearing [54,55,56,57]. These studies suggest that knocking out one connexin will cause the expression levels of other types of connexins to change. The phenotype of *Gjb6*-related deafness models can be caused by the combined effect of the above-mentioned connexin changes.

Because large deletions in the *GJB2* or *GJB6* gene exhibit a digenic mode of inheritance in the induction of deafness in humans, double Cx26^+/−^/Cx30^+/−^ heterozygous mice have been established for mechanism research. These mice show hearing loss with normal development and no apparent hair cell degeneration, but reduced endocochlear potential (EP). However, single Cx26^+/−^ or Cx30^+/−^ heterozygous mice have normal hearing [59]. These data indicate that large deletions in the *GJB2* or *GJB6* gene may impair heterozygous coupling of Cx26 and Cx30, which results in hearing loss though EP reduction.

In addition to Cx26 and Cx30 transgenic mice, other strains including Cx29^−/−^, Cx29^lacZ/lacZ^, and *Gjb3*^−/−^ have also been reported. Cx29^−/−^ mice reported by Tang et al. show early high-frequency hearing loss, prolonged latency of auditory brainstem response (ABR), and severe demyelination of spiral ganglion neurons [65]. Notably, these abnormal changes were not observed in Cx29^lacZ/lacZ^ strains reported by Eiberger et al. [66]. The contradictory outcome of these two studies may be due to the different time points observed by the two teams. *Gjb3*^−/−^ mice show normal morphology and function of the inner ear and skin [52].

Using *Gjb2*-related deafness as an example, the main pathophysiological phenomena of the above deafness models include sensory epithelial cell damage, cochlear developmental disorders, and reduced cochlear amplification and EP. Since Cx26 is not expressed in HCs, the molecular mechanism of hair cell death has not been fully elucidated. In some models, it has been observed that oxidative stress damage and macrophage-related inflammation may be involved in the process of cochlear cell damage [110,111]. Malformed organ of Corti is observed in Cx26 knockout and p.Arg75Trp models and may be caused by cytoskeleton disorder in pillar cells (PCs) [99,106]. In cell-specific Cx26-null models, the impaired DPOAE and changes in OHC nonlinear capacitance suggest that cochlear amplification is reduced, which may be the cause of deafness [48,109]. However, in Cx26 p.Arg75Trp mouse models, the OHC nonlinear capacitance is preserved despite impaired DPOAE [112]. These data indicate that a null mutation in Cx26 or p.Arg75Trp mutation may have different effects on OHC nonlinear capacitance. EP reduction remains a controversial cause of *Gjb2*-related deafness. One study suggested that deafness induced by Cx26 deficiency is not determined by EP reduction. In this study, data showed that the EP could still remain at a high level, even as some Cx26 KO mice exhibited complete deafness [104]. However, another group observed a significant reduction in EP with a small square deviation in Sox10Cre;Cx26 ^flox/flox^ mice [113]. Because connexin mutations can lead to abnormal gap junction function, the above pathological changes may be caused by disorders of intercellular communication. Impaired transfer of glucose, Inositol 1,4,5-trisphosphate (IP3), adenosine triphosphate (ATP), Ca^2+^, and miRNA has been found in connexin-deficient mice or with the use of gap junction channel/hemichannel blockers [114,115,116]. Despite the support of numerous in vitro studies, the deafness mechanisms and pathology of different connexin mutations need to be carefully interpreted. For example, impaired glucose transfer was observed in both a Cx30 null model and *Gjb2*^+/−^ mice [111,115]. This evidence suggests that glucose transport is also impaired in Cx26 knockout mice. This leads to the question as to whether glucose deficiency could be the cause of developmental disorders of the organ of Corti (OC) in Cx26-null mice. The answer appears to be no because the Cx30 knockout mice did not exhibit OC dysplasia. Furthermore, some connexin mutations can affect protein expression of other connexins, which leads to the disruption of gap junction plaques [54,55,117]. In future studies, it is necessary to consider the interaction of various connexins and further explore the mechanisms of these different pathological phenomena.

### 2.2. Congenital Cataract

#### 2.2.1. Connexin Mutations and Congenital Cataract

Congenital cataract has a prevalence of 1–6 per 10,000 live births in developed countries and 5–15 per 10,000 live births in developing countries. Approximately 50% of cases may have a genetic cause, and *GJA8* (encoding Cx50) and *GJA3* (encoding Cx46) gene mutations account for about 20% of non-syndromic inherited cataract cases [118,119,120]. To date, over 90 mutations in the *GJA8* or *GJA3* gene have been reported, mainly in cases of autosomal dominant inheritance. Only four mutations (c.776insG, c.670insA, c.649G>A, and c.89dupT) in the *GJA8* gene are found in autosomal recessive inheritance (http://www.hgmd.cf.ac.uk/ac/index.php (accessed on 8 April 2022)) [121,122,123,124].

Cx46 and Cx50 are highly co-expressed in fiber cells and differentiated epithelial cells of the equatorial region of the mammalian lens. Furthermore, Cx50 is separately expressed in epithelial cells [28,41,125,126]. Differences in the expression patterns of Cx46 and Cx50 may be the reason that *GJA3* gene mutations cause cataracts only, while *GJA8*-related cataracts are accompanied with microcornea or glaucoma in half of the reported cases [127]. Congenital cataracts caused by *GJA8* and *GJA3* gene mutations show a variety of phenotypes, including nuclear, perinuclear, zonular, punctiform, pulverulent, jellyfish-like, star-shaped, full moon, Y-sutural, balloon-like, lamellar, and triangular phenotypes [121,127]. The various phenotypes indicate that different gap junction gene mutations may result in distinct pathogenic mechanisms of congenital cataracts. To date, the in vivo mechanism of connexin mutations that cause congenital cataracts has not been illustrated, as knock-in animal models are limited. In the following section, we summarize the known knock-in and knockout animal models and analyze possible pathophysiology, which may be beneficial to further mechanism research in the future.

#### 2.2.2. Mouse Models for Human Congenital Cataract

To date, only five Cx50 mutation mouse strains (Cx50^+/V64A^, Cx50^+/G22R^, Cx50^+/R205G^, Cx50^+/D47A^, and Cx50^+/S50P^) and one Cx46fs380 knock-in strain have been reported. All of these strains develop dominant cataracts [30,35,36,37,38,39,128,129]. In vivo, these mutations act as gain-of-function mutations that affect the protein levels of co-expressed connexins (e.g., reduced Cx46 protein level in the lens of Cx50^+/D47A^ mice [130] or reduced Cx50 protein level in lens of Cx46fs380 knock-in mice [30]) or disrupt phosphorylated states of co-expressed connexins (e.g., decreased level of phosphorylated Cx46 in the lens of Cx50^+/G22R^ [36] or Cx50^+/R205G^ mice [37]). Gao et al. found that both Cx50 p.Gly22Arg and Cx50 p.Arg205Gly mutations in vivo require interaction with endogenous wide-type (WT) Cx46 to disrupt lens peripheral fiber cells, indicated by the observation that disrupted peripheral fiber cells with vacuoles or enlarged extracellular spaces were only found in the lenses of *Gja8*^G22R/G22R^*Gja3*^+/+^ and *Lop10*/*Lop10* mice but not in *Gja8*^G22R/G22R^*Gja3*^−/−^ and *Lop10/Lop10*α3^−/−^ mice [36,37]. Moreover, another in vivo study revealed a different effect on lens fiber cell formation when Cx50 p.Ser50Pro interacts with WT Cx50 or WT Cx46 only. This study found that *Gja8*^S50P/−^*Gja3*^+/+^ mice (in which Cx50 p.Ser50Pro interacts with WT Cx46 only) show disruption of secondary fiber cells as in *Gja8*^G22R/G22R^*Gja3*^+/+^ and *Lop10*/*Lop10* mice, while *Gja8*^S50P/+^*Gja3*^−/−^ mice (in which Cx50 p.Ser50Pro interacts with WT Cx50 only) specifically display impaired elongation of primary fiber cells with normal peripheral cortical fibers [39].

It has been suggested that Cx46 is essential for lens transparency as well as lens stiffness, while Cx50 is important for lens growth. The Cx50 mutation mice and Cx50 knockout mice mentioned above all developed cataracts accompanied by small lenses, while Cx46fs380 knock-in mice and Cx46 knockout mice exhibit normal lens size [28,30,35,36,37,39,40,41,130,131]. Additionally, White et al. found that targeted replacement of Cx50 with Cx46 in Cx50KI46 mice can prevent cataracts, but these mice still exhibit microphthalmia [42]. Cx50 knockout and mutation mice exhibit disruption of fiber cell formation and epithelial cell proliferation [35,36,37,39,130,132]. Impaired denucleation, impaired degradation of mitochondria, and reduced β-crystallins are found in the lenses of Cx50^+/D47A^ mice [130]. In addition, altered solubility of crystallin and delayed denucleation processes have been observed in the lenses of Cx50 knockout mice, and the severity of cataracts is dependent on the differentially altered solubility of crystallin proteins [40,41,131]. Cx46 knockout mice (α3^−/−^) and Cx46fs380 knock-in mice develop nuclear cataracts with normal differentiation of lens fibers. These mice show aberrant proteolysis of crystallins and increased insolubility of some crystallins [28,30]. In addition, Gong et al. recently found that deletion of Cx46 increases lens stiffness in old mice with C57BL/6J and 129SvJae strain backgrounds as well as in young mice with the C57BL/6J strain background [133].

In conclusion, disruption of fiber cell differentiation and formation as well as decreased epithelial cell proliferation may be the pathophysiology of congenital cataracts and microphthalmia caused by connexin mutations. These pathophysiologic changes include abnormal morphology of lens fiber cells and epithelial cells, aberrant solubility and proteolysis of some crystallins, and impaired denucleation and degradation of organelles. Furthermore, Cx50 and Cx46 mutations exhibit gain-of-function to interact with WT or other co-expressed connexins in vivo, which results in congenital cataracts. The interdependence and interaction of connexins have also recently been revealed in the testicular response to insulin [134]. However, it remains unclear how Cx50 and Cx46 mutations regulate the pathophysiologic process in vivo.

Cx46 is known to be a pro-tumorigenic factor in multiple tumor types and has been shown to play a role in cancer stem cell (CSC) proliferation, survival, and self-renewal in glioblastoma, and enhancement of CSC and cancer aggressiveness in melanoma and breast cancer. The C-terminus of Cx46 mediates protein–protein interactions (e.g., interaction with Nopp-140), and Cx46-dependent channel action plays a key role in tumorigenesis [1,135,136]. Elucidation of the pathogenic mechanism in tumors may provide additional insight into cataractogenesis.

### 2.3. Congenital Heart Diseases

#### 2.3.1. Connexin Mutations and Congenital Heart Diseases

Gap junctions exist widely in cardiomyocytes and non-cardiomyocytes and are essential for impulse conduction in the cardiac conduction system and maintenance of normal cardiac function. Cx43 is the most abundant connexin expressed in cardiomyocytes, fibroblasts, and endothelial cells of the mammalian heart [137,138]. Cx40 is mainly expressed in mammalian atrial myocytes and the cardiac conduction system [138]. To date, over 50 mutations in the *GJA1* (encoding Cx43) and *GJA5* (encoding Cx40) genes have been reported to be associated with heart disease (http://www.hgmd.cf.ac.uk/ac/index.php (accessed on 8 April 2022)). *GJA1* gene mutations, which cause heart disease, often correspond with cardiac malformations (e.g., ventricular septal defect [VSD], atrioventricular septal defect [AVSD], and hypoplastic left heart syndrome [HLHS]) in mainly autosomal recessive inheritance [139,140,141]. Notably, a *GJA1* gene mutation (p.Glu42Lys) leads to sudden infant death syndrome (SIDS) without cardiac malformations. Immunostaining of cardiac tissue from a patient with a p.Glu42Lys mutation showed mosaic loss of Cx43 [142]. For *GJA5* gene mutations, different types of mutations result in different heart diseases. Missense or nonsense mutations mainly cause atrial fibrillation through autosomal dominant inheritance, while gross deletions and insertions lead to cardiac malformations, most of which are tetralogy of Fallot (TOF) malformations (http://www.hgmd.cf.ac.uk/ac/index.php (accessed on 8 April 2022)) [143,144,145,146,147]. In addition to *GJA1* and *GJA5*, one mutation (p.Arg75His) in the *GJC1* (encoding Cx45) gene has been reported to be related to heart disease to date [148]. Although many functional in vitro experiments have revealed impaired trafficking and assembly, disruption of connexin phosphorylation, and aberrant permeability and electrical coupling of gap junctions caused by connexin mutations (described in the Section 3 of this review), pathophysiological changes have not been illustrated in vivo. Therefore, knockout and knock-in animal models are crucial to mechanism research in vivo. The available animal models are described in the following section.

#### 2.3.2. Mouse Models for Human Congenital Heart Diseases

##### *Gja1*-Related Heart Disease Models

The C-terminus of Cx43 contains multiple phosphorylation sites that regulate trafficking, assembling of Cx43, protein turnover, and gap junctional communication. The C-terminus is also a crucial domain for interactions between Cx43 and partners such as protein zonula occludens 1 (ZO-1) [149,150,151]. Association with heart disease has been reported in two Cx43 mutant mouse models (Cx43^K258stop^ and α-MHC-Cre;Cx43^floxD378stop/floxD378stop^) that lack the C-terminal region of Cx43. Cx43^K258stop^ mice show left ventricular dilation, atrioseptal defect, and a QT-prolongation, and mainly die shortly after birth due to an epidermal barrier defect [24]. However, α-MHC-Cre;Cx43^floxD378stop/floxD378stop^ mice die from severe ventricular arrhythmias with no obvious cardiac malformations and normal gap junctional coupling. Decreases in sodium and potassium current densities and loss of Nav1.5 protein may be the cause of ventricular arrhythmias [25]. This study was the first to reveal that Cx43-related arrhythmias can occur without impairment of gap junctional function. In addition to deletion of the C-terminus, recent research by Xiao et al. has revealed a novel in vivo mechanism of forward trafficking of Cx43 to the cell membrane [152]. Xiao et al. created a Cx43^M213L^ mouse model in which Cx43 p.Met213Leu mutation led to generation of full-length Cx43, but not *GJA1*-20k, which is the alternative translation of Cx43 mRNA and an N-terminal truncation of the full protein. Homozygous Cx43^M213L^ mice show reduced expression of full-length Cx43 and gap junctions, which indicates that *GJA1*-20k is needed for Cx43 forward trafficking in the heart.

Some studies have revealed that disruption of phosphorylated Cx43 is associated with arrhythmias rather than cardiac malformations. Cx43^+/I130T^ and α-MHC-Cre;Cx43^+/floxG138R^ mice both show loss of phosphorylated forms of Cx43. α-MHC-Cre;Cx43^+/floxG138R^ mice exhibit spontaneous arrhythmias and α-MHC-Cre;Cx43^+/floxG138R^ mice have increased susceptibility to arrhythmias [22,26]. Cx43^S282A+/−^ mice, in which the mutation results in Cx43-serine 282 dephosphorylation show cardiomyocyte apoptosis and ventricular arrhythmias. Furthermore, Sun et al. found that the Cx43^S282A^ mutation also blocks phosphorylation of Cx43-serine S279 and impairs gap junction coupling [153,154]. Huang et al. found that two *Gja1* knock-in mouse models (*Gja1*^S368A^ and *Gja1*^S325A/S328Y/S330A^) that feature mutations at important phosphorylation sites have no overt heart malformations [155].

Cx43 expression in different regions has distinct effects on heart morphogenesis and physiology. Three Cx43 knockout (Cx43^−/−^, P3pro-Cre;Cx43^flox/flox^, and Wnt1-Cre;Cx43^flox/flox^) mouse models and one overexpression (CMV43) mouse model all result in abnormal coronary deployment. Additionally, each of these models, except for the Wnt1-Cre;Cx43^flox/flox^ strain, show malformation of the cardiac outflow tract (OFT) [15,16,18]. As Cx43 is specifically knocked out in the cardiac neural crest (CNC) of P3pro-Cre;Cx43^flox/flox^ and Wnt1-Cre;Cx43^flox/flox^ mice, it has been suggested that Cx43 expression in CNC is essential to normal coronary development, but not to normal formation of OFT. Moreover, two cardiomyocyte-specific knockout strains (α-MHC-Cre;Cx43^flox/flox^ and MLC2v-CreCx43^flox/flox^) show normal cardiac structure and contractile function, but sudden arrhythmic death occurs within two months after birth [17]. This suggests that Cx43 expression in cardiomyocytes is essential to maintenance of normal heart rhythm but is not associated with cardiac malformations at birth. However, the role of Cx43 in non-cardiomyocytes has not been revealed clearly by three non-cardiomyocyte-specific mouse models (Cx43fsp1KO, VEC Cx43 KO, and TIE2-Cre;Cx43^flox/−^) [156,157,158].

##### *Gja5*- and *Gjc1*-Related Heart Disease Models

Cx40^A96S^ mice, in which the mutation results in loss-of-function of gap junctions, exhibit induced atrial fibrillation in the absence of overt fibrosis of atrial tissue [159]. Cx40^−/−^ mice exhibit various types of cardiac malformations including ventricular septal defect, TOF, double-outlet right ventricle (DORV) and endocardial cushion defects, atrial arrhythmias, and cardiac conduction abnormalities of the atrioventricular block and bundle branch block [31,32,33].

The role of Cx45 in cardiomyocytes has been revealed by germline knockout (Cx45^−/−^) and cardiomyocyte-specific knockout (α-actin-Cre;Cx45 ^flox/flox^) mice. Both of these mouse models show embryonic lethality and conduction block. However, Cx45^−/−^ mice have endocardial cushion defects, while α-actin-Cre;Cx45 ^flox/flox^ mice do not [60,61]. These findings suggest that Cx45 expression in cardiomyocytes is essential to embryonic survival, but not to endocardial cushion formation. However, replacement of Cx45 with Cx36 in cardiomyocytes results in not only embryonic lethality but also endocardial cushion defect [62], which suggests that ectopic Cx36 in cardiomyocytes also impairs the formation of endocardial cushion. Furthermore, conditional knockout mice (α-MHC-CreER;Cx45^flox/flox^) revealed that Cx45 is involved in atrioventricular nodal conduction in adult mice [64]. Moreover, Cx45 overexpression in cardiomyocytes increases the susceptibility of ventricular arrhythmias in Cx45OE mice [63].

To summarize, cardiac malformations and arrhythmias are the common inherited congenital heart diseases caused by connexin mutations. The knockout and knock-in mouse models described above reveal that integrity of the C-terminus, alternative translation of mRNA, phosphorylation states, and correct expression regions and patterns of connexins play a vital role in embryonic survival and heart development through the regulation of connexin assembly and trafficking, which affects gap junctional function and sodium and potassium currents. These findings indicate that there must be shared and differential functions among different connexins in the heart because replacement of Cx43 with Cx32 and Cx40 can prevent the death of mice caused by Cx43 deletion, while replacement of Cx43 with Cx26 and Cx31 cannot [19,20,21]. Moreover, replacement of Cx40 with Cx45 can rescue the conduction abnormalities in Cx40^−/−^ mice [34]. Thus, an understanding of the pathophysiology of congenital heart diseases may aid in the study of the pathogenic mechanisms of other hereditary diseases caused by connexin mutations.

### 2.4. Hereditary Skin Diseases

#### 2.4.1. Connexin Mutations and Hereditary Skin Diseases

Because the epidermis does not have a blood supply, gap junctions that mediate intercellular communication are essential to the physiology of the skin [160]. Cx26 (encoded by *Gjb2*) and Cx30 (encoded by *Gjb6*) are widely expressed not only in the inner ear of mammals, but also in the epidermis of the skin where Cx43 (encoded by *Gja1*), Cx30.3 (encoded by *Gjb4*), and Cx31 (encoded by *Gjb3*) are also expressed. In addition, Cx43 is expressed in the dermis and hypodermis of mammalian skin [160,161,162].

Syndromic deafness caused by *GJB2* gene mutation is often accompanied by skin diseases, including keratitis–ichthyosis–deafness (KID) syndrome [51], Bart–Pumphrey syndrome (BPS) [163], hystrix-like ichthyosis with deafness (HID) syndrome [164], and Vohwinkel syndrome [165]. Among them, KID syndrome is the most common disease. To date, over 20 mutations in the *GJB2* gene have been reported to be associated with skin diseases (http://www.hgmd.cf.ac.uk/ac/index.php (accessed on 8 April 2022)). All of the skin diseases caused by *GJB2* gene mutations are commonly related to hyperkeratosis (especially palmoplantar hyperkeratosis) and mainly exhibit autosomal dominant inheritance [166].

For *GJB6* gene mutations, different mutation sites result in distinct diseases, which include non-syndromic deafness and Clouston syndrome (http://www.hgmd.cf.ac.uk/ac/index.php (accessed on 8 April 2022)). Clouston syndrome is an autosomal dominant disease characterized by alopecia, nail dystrophies, and palmoplantar hyperkeratosis [166]. This syndrome is not commonly associated with deafness. However, two mutations (p.Asn54Lys and p.Gly59Arg) in the *GJB6* gene have recently been reported to result in palmoplantar keratoderma with hearing loss [167,168]. Additionally, a *GJB2* gene mutation (Cx26 p.Asn14Lys) identified in a Dutch child led to a phenotype resembling Clouston syndrome accompanied with deafness [169]. To date, only eight *GJB6* gene mutations have been reported to result in skin diseases, among which six mutations lead to Clouston syndrome (http://www.hgmd.cf.ac.uk/ac/index.php (accessed on 8 April 2022)).

Along with *GJB2* and *GJB6* gene mutations, *GJA1* (encoding Cx43), *GJB4* (encoding Cx30.3), and *GJB3* (encoding Cx31) gene mutations also lead to hereditary skin diseases. In addition to causing deafness and heart diseases, *GJA1* gene mutations mainly result in ODDD. ODDD is an autosomal dominant disease characterized by malformations of the craniofacial bones, abnormalities of the eyes, teeth, skin, hair, and nails, and syndactyly [166]. To date, more than 70 mutations in the *GJA1* gene have been reported to result in ODDD (http://www.hgmd.cf.ac.uk/ac/index.php (accessed on 8 April 2022)). *GJB4* and *GJB3* gene mutations can result in erythrokeratodermia variabilis (EKV) [170], and over 20 mutations in the *GJB4* and *GJB3* genes have been reported to be associated with EKV.

Although a variety of in vitro functional studies of Cx26, Cx30, and Cx43 mutations have been carried out and various possible pathogenic changes (described in the Section 3 of this review) have been revealed, it remains unclear why some Cx26 and Cx30 mutations cause deafness without skin diseases in vivo but others induce syndromic deafness accompanied with skin changes. Similarly, it is unclear why some Cx43 mutations lead to human ODDD but others result in deafness or cardiac changes only. Because knock-in animal models are limited, further research is needed. Available mouse models for the study of human hereditary skin diseases are described in the following section.

#### 2.4.2. Mouse Models for Human Hereditary Skin Diseases

To date, only seven mutation mouse models associated with hereditary skin diseases have been reported. The K10Cx26 (D66H) mouse model mimics human Vohwinkel syndrome [50]. The pgk-Cre;Cx26^+/floxS17F^ and Cx26-G45E mouse models mimic human KID syndrome [44,51]. The pgk-Cre;Cx30^+/floxA88V^ mouse model mimics human Clouston syndrome [58]. The pgk-Cre;Cx31^+/floxF137L^ mouse model mimics human EKV [53]. The Cx43^+/jrt^ and pgk-Cre;Cx43^+/floxG138R^ mouse models both mimic human ODDD [26,27].

It is notable that ODDD-like phenotypes are found in heterozygous Cx43^+/jrt^ mice but not in heterozygous Cx43 null mice [15,171]. The K10Cx26 (D66H), Cre;Cx26^+/floxS17F^, Cx26-G45E, and pgk-Cre;Cx30^+/floxA88V^ mice all show obvious epidermal hyperkeratosis, while homozygous loss of Cx26 or Cx30 (in humans or mice) results in no clinically discernible epidermal abnormality [44,50,51,88,172,173,174]. These findings indicate that connexin mutations associated with hereditary skin diseases are not simple loss-of-function mutations in vivo. The dominant negative or trans-dominant negative effect of Cx43^jrt^ and Cx26 p.Asp66His mutations has been identified in vivo [27,50]. However, it is still unclear how these gain-of-function mutations lead to pathophysiological changes in hereditary skin diseases in humans and mice. Further research is needed to determine the signaling pathways involved in the pathogenic processes.

### 2.5. X-Linked Charcot–Marie–Tooth Disease

#### 2.5.1. Connexin Mutations and X-Linked Charcot–Marie–Tooth Disease

Charcot–Marie–Tooth (CMT) disease is the most common inherited neurological disorder, with a prevalence of about 1 in 2500 births around the world. Among the varied phenotypes of CMT, the most common form, X-linked CMT (CMT1X), is caused by *GJB1* (encoding Cx32) gene mutations [175,176]. The *GJB1* gene is expressed in Schwann cells and restricted to Schmidt–Lantermann incisures and the paranodal segment of the nodes of Ranvier in the peripheral nervous system [177]. To date, over 400 mutations in the *GJB1* gene have been reported to cause CMT1X (http://www.hgmd.cf.ac.uk/ac/index.php (accessed on 8 April 2022)). Most of these mutations are missense/nonsense mutations and seem to mainly result in the loss of gap junctional function. Additionally, several Golgi-retained CMT1X mutations (e.g., Cx32 p.Arg75Trp, Cx32 p.Met93Val, and Cx32 p.Asn175Asp) display a dominant negative effect on WT Cx32 [176,178]. There is no specific correlation between phenotypes of CMT1X and different mutations in the *GJB1* gene. However, as CMT1X occurs through X-linked inheritance, the phenotype observed in male patients with CMT1X is usually more severe than the phenotype observed in female patients [179].

Recent studies by Kleopa et al. report that intrathecal gene therapy provides significant therapeutic benefits in Cx32-null and Cx32 T55I KO mouse models, but no improvement in Cx32 R75W KO and Cx32 N175D KO mice [176,180,181]. These findings suggest that there must be distinct pathogenic mechanisms among the different mutations that have not be illuminated. Mechanism-based therapy may be more beneficial to patients with mutations such as Cx32 p.Arg75Trp and p.Asn175Asp. Thus, we summarize available animal models of CMT1X and analyze pathophysiological changes found in these models in the following section.

#### 2.5.2. Mouse Models for Human CMT1X

In addition to the Cx32 null and Cx32 mutation mouse models mentioned above, a Cx32 R142W KO mouse model was also generated for pathogenic mechanism research of CMT1X. All of these *Gjb1*-related CMT1X mice develop progressive demyelinating peripheral neuropathy marked by thin myelin sheaths, supernumerary Schwann cells, axonal degeneration, and enlarged periaxonal collars [180,182,183,184,185]. Additionally, an increased number of macrophages in demyelinating nerves, increased expression of chemokine monocyte chemoattractant protein-1 (MCP-1), and macrophages in contact with degenerating myelin are found in Cx32-deficient mice [183,186].

Currently, multiple studies have revealed that secondary inflammation involving macrophages is involved in the pathogenic process of CMT1X. By cross breeding Cx32 null mice with MCP-1 knockout mice, Groh et al. found that attenuation of MCP-1, a chemokine related to the recruitment of macrophages, results in reduced macrophage numbers and improvement of demyelination in Cx32-deficient mice. Groh et al. also found that MEK–ERK signaling regulates the increased expression of MCP-1 in Schwann cells of Cx32 null mice [186]. Furthermore, Groh et al. found that cytokine colony-stimulating factor 1 (CSF-1)-activated macrophages are strongly related to the dedifferentiation of Schwann cells when the loss of Cx32 occurs in vivo, as CSF-1/Cx32 double knockout mice show amelioration of demyelination with no upregulation of NCAM and L1 (Schwann cell dedifferentiation markers) [187]. An additional study from Groh et al. in 2016 revealed that different isoforms of CSF-1 play a distinct role in macrophage-mediated peripheral neuropathy in Cx32 null mice. The study demonstrated that the secreted proteoglycan isoform of CSF-1 mediates macrophage-related neural damage, while the cell-surface isoform of CSF-1 attenuates peripheral neuropathy [188].

Overall, macrophage-mediated demyelination of peripheral nerves may be the crucial pathophysiology of CMT1X, and MCP-1, CSF-1, and MEK–ERK signaling have been found to be involved in this process. Recently, Kagiava et al. and Scherer et al. identified that Cx32 mutations Cx32 p.Arg75Trp, p.Asn175Asp, and p.Arg142Trp exhibit dominant negative effects on WT Cx32 in vivo and no significant improvement of peripheral neuropathy caused by these mutations was achieved through intrathecal gene therapy [176,180,181,189]. Thus, mechanism-based therapy is vital to patients with mutations such as Cx32 p.Arg75Trp, p.Asn175Asp, and p.Arg142Trp. Intervention of the macrophage activation and recruitment process may be useful to prevent CMT1X caused by Cx32 mutations, although further molecular mechanism research is needed.

## 3. Functional Effects of Gene Mutations on Corresponding Connexins

**Figure 2:**
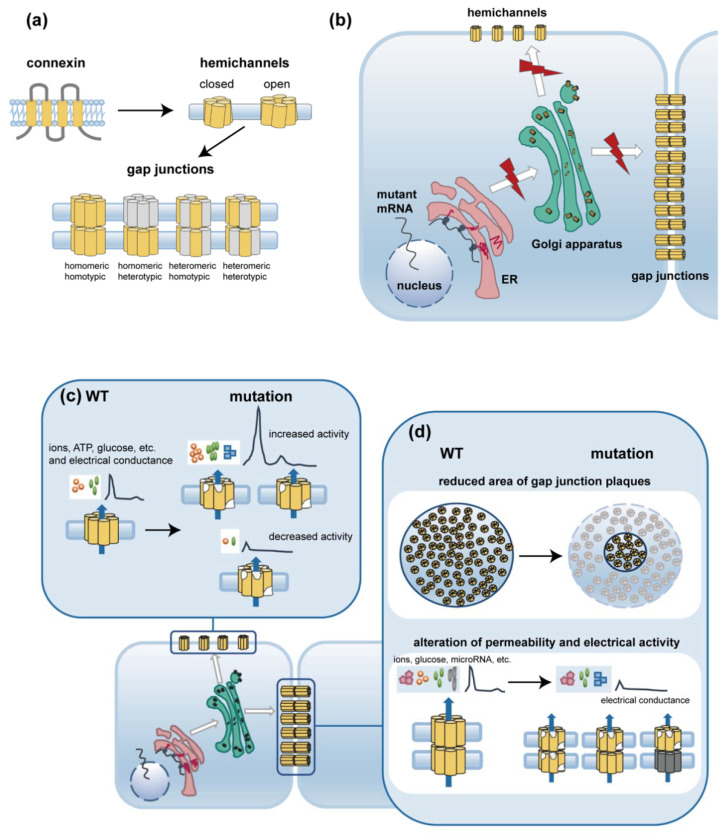
Diagram of possible pathogenesis of gap junction disorders caused by gene mutations. (**a**) structure of connexin, hemichannels, and gap junctions; (**b**) Gene mutations lead to the disruption of translation, assembly, and trafficking of connexins, which results in a loss of gap junction or hemichannel formation; (**c**,**d**) Gene mutations reduce the area of gap junction plaques and impair the function of gap junctions and hemichannels. (**c**) Gene mutations result in aberrant channel permeability and electric activity of hemichannels. (**d**) Gene mutations result in reduced area of gap junction plaques, altered channel permeability, and abnormal electric activity of gap junctions. WT: wild-type. ER: endoplasmic reticulum, ATP: adenosine triphosphate.

A variety of functional in vitro experiments have been performed to explore the pathogenesis of hereditary diseases caused by connexin mutations. Here, we analyze and summarize the shared functional effects of different mutations in inner ear, eye, heart, and skin-expressed gap junction genes (Figure 2).

### 3.1. Effects on Hemichannel or Gap Junction Formation

Most mutants fail to form hemichannels or gap junctions as follows: (1) Some mutants (e.g., Cx26 c.35delG, Cx26 p.Met1Val, Cx26 p.Phe161Ser, and Cx26 p.Pro173Arg) result in no protein being detected, although mRNA is synthesized [190,191]. (2) Some mutants (e.g., Cx26 p.Gly12Val, Cx26 p.Trp77Arg, Cx43 p.Phe52dup, Cx43 p.Arg202His, Cx40 p.Pro88Ser, and Cx50 p.Asp47Asn) show impaired trafficking. These mutations are entirely retained in the endoplasmic reticulum (ER), Golgi apparatus, or other intracellular compartments without being correctly targeted to the cell membrane [143,190,192,193,194]. (3) Although some mutants (e.g., Cx26 p.Ser19Thr, Cx26 p.Leu90Pro, CX50 p.Arg23Thr, Cx43 p.Tyr17Ser, Cx43 p.Ala40Val, Cx43 p.Ile130Thr, and Cx40 p.Gly38Asp) display plasma membrane localization, they form reduced gap junction plaques [143,190,194,195]. (4) Some mutants (e.g., Cx26 p.Asn14Tyr, Cx26 p.Ser17Phe, Cx26 p.Met34Thr, Cx26 p.Leu90Pro, Cx26 p.Arg184Pro, and Cx26 p.Trp44Cys) have been confirmed to partly or completely lose the ability to form homomeric hemichannels by oligomerization studies [191,193,196].

### 3.2. Functional Effects of Gene Mutations on Gap Junctions

Although some mutants are correctly synthesized and localized in cell membranes to form gap junction plaques, gap junctional functions may be aberrant in the following ways: (1) Gap junctions formed by some mutants (e.g., Cx26 p.Met34Thr, Cx26 p.Val84Leu, Cx43 p.Tyr17Ser, Cx43 p.Gly21Arg, Cx43 p.Ala40Val, Cx43 p.Phe52dup, Cx43 p.Leu90Val, Cx43 p.Ile130Thr, and Cx50 p.Arg23Thr) display impaired permeability with reduced intercellular transfer of dye, ions, or metabolic products [195,197,198,199]. (2) Some mutants result in abnormal electrical behavior. For example, the Cx43 p.Tyr17Ser mutation results in a complete lack of electric coupling, as no unitary or junctional conductance was recorded in transfected N2A cells [194]. The mutation Cx43 p.Ile130Thr demonstrates a significantly reduced junctional conductance even with a similar unitary conductance compared with WT Cx43. In addition, this mutation shows slower conduction velocity [22,194].

Additionally, some mutants (e.g., Cx26 p.Trp44Cys, Cx26 p.Trp44Ser, Cx26 p.Arg143Gln, Cx26 p.Asp179Asn, Cx26-Arg184Gln, Cx26-Cys202Phe, Cx26 p.Gly59Ala, Cx26 p.Arg75Gln, Cx26 p.Arg75Trp, Cx43 p.Arg202His, Cx43 p.Gln49Lys, Cx43 p.Leu90Val, Cx43 p.Val216Leu, Cx40 p.Pro88Ser, Cx40 p.Ala96Ser, CX50 p.Arg23Thr, Cx50 p.Glu48Lys, Cx46 p.Asp3Tyr, and Cx46 p.Leu11Ser) have dominant or trans-dominant negative effects on co-expressed wild-type connexins that lead to the reduced area of gap junction plaques and decreases in dye coupling or electrical coupling [143,195,200,201,202,203].

### 3.3. Functional Effects of Gene Mutations on Hemichannels

In addition to their incorporation into gap junctions, hemichannels play important roles in allowing communication between cells and the extracellular environment. Although some mutants can be synthesized and correctly targeted to the plasma membrane to form hemichannels, the channel properties are aberrant, which results in enhanced or impaired hemichannel function. Some mutants (e.g., Cx26 p.Gly45Glu, Cx26 p.Asp50Ala, Cx26 Ala88Val, Cx30 Gly11Arg, Cx30 p.Ala88Val, Cx43 p.Gly138Arg, Cx43 p.Gly143Ser, Cx40 p.Val85Ile, Cx40 p.Leu221Ile, Cx46 p.Thr19Met, and CX50 p.Gly46Val) form homomeric hemichannels with significantly increased dye uptake, membrane current flow, or release of ATP compared with wild-type connexins [204,205,206,207,208,209,210,211]. Some mutants (e.g., Cx26 p.His73Arg, Cx26 p.Ser183Phe, Cx26 p.Gly12Arg, Cx26 p.Asn14Tyr, Cx26 p.Ser17Phe, and Cx50 p.Gly46Val) form hyperactive heteromeric hemichannels that co-express with wild type connexins [196,212,213]. Some mutants (e.g., Cx26 p.Glu47Lys, Cx43 p.Leu90Val, Cx43 p.Ile130Thr, Cx50 p.Trp45Ser, Cx50 p.Ser276Phe, Cx50 p.Val44Ala, Cx46 p.AspD3Tyr, and Cx46 p.Leu11Ser) form homomeric hemichannels that exhibit less dye uptake and membrane current flow than hemichannels formed by wild type connexins [199,202,209,213,214,215,216].

## 4. Conclusions

Connexin-formed gap junctions are found in a variety of organs and tissues and act to mediate intercellular communication and maintain normal physiological function. Mutations in gap junction genes are related to hereditary diseases including hereditary deafness, congenital cataract, congenital heart diseases, hereditary skin diseases, and CMT1X. A large number of knockout and knock-in animal models have been used to study the pathology and pathogenesis of these diseases. We have identified some similarities among the pathology and pathogenesis of different hereditary diseases in these animal models.

Disruption of cellular proliferation and differentiation has been identified in different connexin-deficient or mutation mice, which indicates that connexins are essential to organ development. Cytoskeletal disorder in PCs is observed in Cx26 knockout and p.Arg75Trp mouse models and may be the cause of the malformed organ of Corti seen in these models [99,106]. Disruption of fiber cell differentiation (impaired denucleation and degradation of mitochondria) and epithelial cell proliferation has been identified in Cx50 knockout and mutation mice [35,36,37,39,130,132]. Cardiac malformations including atrioseptal defect, TOF, DORV, and endocardial cushion defects are found in Cx43- or Cx40-deficient or mutation mouse models [15,16,18,31,32,33]. Altered epidermal proliferation and differentiation occur in K10Cx26 (D66H), Cre;Cx26^+/floxS17F^, Cx26-G45E, and pgk-Cre;Cx30^+/floxA88V^ mice, which results in epidermal hyperplasia and hyperkeratosis [44,50,51]. In addition, dedifferentiation of Schwann cells is observed in Cx32 null mice [187].

Protein interactions between co-expressed connexins have been identified in various connexin deficient or mutation mice and play a vital role in the onset of different hereditary diseases. Some connexin mutations act as gain-of-function mutations in vivo that affect protein levels of co-expressed connexins (e.g., decreased protein level of Cx26 in the cochlea of Cx30^T5M/T5M^ mice [54], reduced Cx46 protein level in the lens of Cx50^+/D47A^ mice [130], reduced Cx50 protein level in the lens of Cx46fs380 knock-in mice [30], and reduction of WT Cx43 in the heart of heterozygous Cx43^+/jrt^ mice [27]). Some mutations in vivo disrupt phosphorylated states of co-expressed connexins (e.g., decreased level of phosphorylated Cx46 in the lens of Cx50^+/G22R^ [36] or Cx50^+/R205G^ mice [37], loss of phosphorylated forms of Cx43 in the heart of heterozygous Cx43^+/I130T^ or α-MHC-Cre;Cx43^+/floxG138R^ mice [22,26]). It has been reported that phosphorylation of connexins is associated with assembling and trafficking [149,150,151]. Thus, reduced phosphorylation may result in the disruption of gap junction or hemichannel formation. In K10Cx26 (D66H) mice, Cx26 Asp66His mutation leads to a loss of Cx26 and Cx30 in the epidermal keratinocyte membrane and accumulation in the cytoplasm [50]. Gao et al. found that Cx50 mutations in vivo require interaction with endogenous WT Cx46 to disrupt peripheral fiber cells in the lens [36,37]. Kagiava et al. and Scherer el al. found that Cx32 mutations p.Arg75Trp, p.Asn175Asp, and p.Arg142Trp exhibit dominant negative effects on WT Cx32 in vivo, which suggests that patients with these dominant mutations receive no benefits from gene therapy [176,180,181,189].

In addition, macrophage-related cochlear cell damage in Cx26 knockout mice and macrophage-mediated demyelination of peripheral nerves in Cx32 null mice indicate that secondary inflammation involving macrophages may be involved in the pathogenic processes of different hereditary diseases, which provides new insight regarding the pathogenesis of congenital cataract, congenital heart diseases, and hereditary skin diseases.

Although various cellular mechanisms of connexin mutations have been revealed, including a complete loss of gap junctional functions, altered channel permeability (ions, ATP, IP3, Ca^2+^, glucose, miRNA), and aberrant channel electric activity (e.g., hyperactive hemichannels formed by co-expression of Cx26 with wild type Cx43 [196]), the precise pathogenesis underlying how these connexin mutations cause hereditary diseases is still unclear. Intrathecal gene therapy may provide significant therapeutic benefits in connexin-null mouse models, but no improvement in mice with some dominant mutations. Thus, mechanism-based therapy (e.g., hemichannel inhibitors, flufenamic acid treatment to improve epidermal pathology in a Cx26-G45E mouse model [217]) may be more beneficial. We hope that this review will stimulate new ideas for further research of these hereditary diseases.

## Figures and Tables

**Table 1:** The connexins in human and mice and related hereditary diseases.

Human		Hereditary Diseases	Mice	
Gene Name	Protein Name		Gene Name	Protein Name
*GJA1*	CX43	ODDD; Congenital heart diseases; SIDS; Hereditary deafness.	*Gja1*	Cx43
*GJA3*	CX46	Congenital cataract	*Gja3*	Cx46
*GJA4*	CX37		*Gja4*	Cx37
*GJA5*	CX40	Atrial fibrillation; Congenital heart diseases	*Gja5*	Cx40
*-*	-		*Gja6*	Cx33
*GJA8*	CX50	Congenital cataract	*Gja8*	Cx50
*GJA9*	CX59		*-*	-
*GJA10*	CX62		*Gja10*	Cx57
*GJB1*	CX32	Hereditary deafness	*Gjb1*	Cx32
*GJB2*	CX26	Hereditary deafness; KID syndrome; HID syndrome; BPS; Vohwinkel syndrome	*Gjb2*	Cx26
*GJB3*	CX31	EKV; Hereditary deafness	*Gjb3*	Cx31
*GJB4*	CX30.3	EKV	*Gjb4*	Cx30.3
*GJB5*	CX31.1		*Gjb5*	Cx31.1
*GJB6*	CX30	Hereditary deafness; Clouston syndrome	*Gjb6*	Cx30
*GJB7*	CX25		*-*	-
*GJC1*	CX45	Heart disease	*Gjc1*	Cx45
*GJC2*	CX47		*Gjc2*	Cx47
*GJC3*	CX30.2/CX31.3	Hereditary deafness	*Gjc3*	Cx29
*GJD2*	CX36		*Gjd2*	Cx36
*GJD3*	CX31.9		*Gjd3*	Cx30.2
*GJD4*	CX40.1		*Gjd4*	Cx39
*GJE1*	CX23		*Gje1*	Cx23

Gene and protein names are summarized from https://www.omim.org (accessed on 8 April 2022) and http://www.informatics.jax.org (accessed on 8 April 2022). ODDD: oculodentodigital dysplasia. SIDS: sudden infant death syndrome. KID syndrome: keratitis–ichthyosis–deafness syndrome. HID syndrome: hystrix-like ichthyosis with deafness syndrome. BPS: Bart–Pumphrey syndrome. EKV: erythrokeratodermia variabilis.

**Table 2:** Summarization of related mouse models in text.

Mouse Model	Symptom	Pathophysiological Changes	Reference
Cx43^−/−^	heart disease	obstruction of right ventricular outflow tract and abnormal coronary deployment	[15]
CMV43	heart disease	malformation of the conotruncus	[16]
α-MHC-Cre;Cx43^flox/flox^	sudden arrhythmic death	spontaneous ventricular arrhythmias, reduced ventricular conduction velocity	[17]
MLC2v-Cre;Cx43^flox/flox^	sudden arrhythmic death	spontaneous ventricular arrhythmias	[17]
Wnt1-Cre;Cx43^flox/flox^	heart disease	abnormal development of coronary, normal formation of OFT	[18]
P3pro-Cre;Cx43^flox/flox^	heart disease	infundibular bulging and coronary anomalies	[18]
Cx43KI32	spontaneous ventricular arrhythmias	morphological defects, Spontaneous ventricular arrhythmias	[19]
Cx43KI40	spontaneous ventricular arrhythmias	mild hypertrophy of heart	[19]
Cx43KI31	heart disease	Malformation in the subpulmonary outlet of the right ventricle, low voltage of the QRS complex	[20]
Cx43KI26	heart disease	slowed ventricular conduction	[21]
Cx43^+/I130T^	heart disease	Reduced Cx43 protein level, conduction velocity, and junctional conductance	[22]
*Gja1* ^W45X^	heart disease	Conotruncal malformation, coronary aneurysms.	[23]
Cx43^K258stop^	defect of the heart and the epidermal barrier	Impaired differentiation of keratinocytes, dilatation of the right ventricular outflow tract	[24]
α-MHC-Cre;Cx43^floxD378stop/floxD378stop^	severe ventricular arrhythmias	impaired cardiac sodium and potassium currents	[25]
α-MHC-Cre;Cx43^+/floxG138R^	Spontaneous arrhythmias	loss of the phosphorylated forms of Cx43	[26]
Cx43^+/jrt^	ODDD	dominant-negative effect, syndactyly, enamel hypoplasia, craniofacial anomalies, cardiac dysfunction.	[27]
pgk-Cre;Cx43^+/floxG138R^	ODDD	Syndactyly, enamel hypoplasia, craniofacial, bone and heart anomalies, increased activity of ATP-releasing	[26]
α3^−/−^	late-onset nuclear cataract	proteolysis of crystallins	[28]
α3^−/−^α8^−/−^	cataracts	cell swelling and degeneration of inner fibers, reduction of gamma-crystallin proteins	[29]
Cx46fs380	progressive cataract	Reduced Cx46 protein level, decreased immunoactivity of Cx50	[30]
Cx40^−/−^	atrial arrhythmias	cardiac conduction abnormalities, cardiac malformations	[31,32,33]
Cx40KI45	arrhythmia	increased duration of the P wave, a prolonged and fractionated QRS complex.	[34]
Cx50^+/V64A^	cataract	clefts in the embryonic lens nucleus, abnormal remnants of the fiber cell nuclei	[35]
Cx50^+/G22R^	cataract	a loss-of-function mutant, disruption of the phosphorylated forms of Cx46	[36]
Cx50^+/R205G^	cataracts and microphthalmia	disruption of the phosphorylated forms of Cx46	[37]
Cx50^+/D47A^	cataract	/	[38]
Cx50^+/S50P^	cataract	primary lens fiber cells failed to fully elongate	[39]
Cx50-null	nuclear cataract	microphthalmia, small lenses,	[40]
α8^−/−^	nuclear cataract	microphthalmia, small lenses,	[41]
Cx50KI46	normal lens	microphthalmia	[42]
CAG-Cre;Cx26^+/floxR75W^	deafness	malformation of supporting cells, collapse of tunnel of Corti, degeneration of hair cell	[43]
pgk-Cre;Cx26^+/floxS17F^	deafness, hyperplasia of tail and foot epidermis, wounded tails, annular tail restrictions	reduction of the endocochlear potential	[44]
Otog-Cre;Cx26^LoxP/LoxP^	deafness	cell death of supporting cells and hair cells	[45]
Sox10-Cre;Cx26^LoxP/LoxP^	deafness	degeneration of organ of Corti and SGN	[46]
Pax2-Cre;Cx26^LoxP/LoxP^	deafness	arrested development of the inner ear	[47]
Foxg1-Cre;Cx26^LoxP/LoxP^	deafness	arrested development of the inner ear	[47]
Rosa26-CreER;Cx26^LoxP/LoxP^	deafness	arrested development of the inner ear	[47]
Prox1-CreER;Cx26^LoxP/LoxP^	deafness	reduces active cochlear amplification	[48]
Lgr5-CreER;Cx26^LoxP/LoxP^	late-onset hearing loss	hair cells loss, morphological change of Deiters’ cells	[49]
K10 Connexin 26 (D66H)	keratoderma	marked thickening of the epidermal cornified layers, premature keratinocyte programmed cell death	[50]
Cx26-G45E	hyperkeratosis, scaling, skin folds, and hair loss	hyperplasia, acanthosis, papillomatosis, increased cell size, and osteal plugging, increased hemichannel currents	[51]
*Gjb3* ^−/−^	embryonic lethality	no abnormalities of skin and inner ear in surviving mice	[52]
pgk-Cre;Cx31^+/floxF137L^	skin disease	hyperproliferation of the stratum germinativum	[53]
Cx30^T5M/T5M^	mild hearing loss	Reduced protein levels of Cx30 and Cx26	[54]
BAC^Cx26^;Cx30^−/−^	normal hearing	no cell death of hair cells	[55]
Cx30^−/−^	severe deafness	disappeared endocochlear potential, degeneration of sensory epithelium, reduced protein levels of Cx26	[56]
pgk-Cre;Cx30^flox/flox^	normal hearing	reduced protein levels of Cx26	[57]
pgk-Cre;Cx30^+/floxA88V^	palmoplantar hyperkeratosis, altered hearing profile	hyperproliferative and enlarged sebaceous glands	[58]
Cx26^+/−^/Cx30^+/−^	hearing loss	reduced endocochlear potential	[59]
Cx45^−/−^	heart disease	endocardial cushion defect, conduction block	[60]
α-actin-Cre;Cx45 ^flox/flox^	heart disease	conduction block	[61]
Cx45KI36	heart disease	defects in cardiac morphogenesis and conduction	[62]
Cx45OE	increased susceptibility of ventricular arrhythmias	remodeling of intercellular coupling	[63]
α-MHC-CreER;Cx45^flox/flox^	arrhythmias	Decreased atrioventricular nodal conductivity and Cx30.2 protein level,	[64]
Cx29^−/−^	high-frequency hearing loss	prolonged latency of ABR, severe demyelination of spiral ganglion neurons	[65]
Cx29^lacZ/lacZ^	normal hearing	no abnormalities of myelin sheaths, normal nerve conduction	[66]

ODDD: oculodentodigital dysplasia, OFT: tetralogy of Fallot, ATP: adenosine triphosphate, SGN: spiral ganglion neuron, ABR: auditory brainstem response.

## Data Availability

The data used to support the findings of this study are included within the article.
